# Circulating Microparticles Carry a Functional Endothelial Nitric Oxide Synthase That Is Decreased in Patients With Endothelial Dysfunction

**DOI:** 10.1161/JAHA.112.003764

**Published:** 2013-02-22

**Authors:** Patrick Horn, Miriam Margherita Cortese‐Krott, Nicolas Amabile, Claas Hundsdörfer, Klaus‐Dietrich Kröncke, Malte Kelm, Christian Heiss

**Affiliations:** 1 Division of Cardiology, Pulmonology, and Vascular Medicine, Medical Faculty, University Duesseldorf, Duesseldorf, Germany (P.H., M.M.C.K., M.K., C.H.); 2 INSERM U970, Paris Cardiovascular Research Center, Paris, France (N.A.); 3 Institute for Organic and Macromolecular Chemistry, University Duesseldorf, Duesseldorf, Germany (C.H.); 4 Institute for Biochemistry and Molecular Biology, Medical Faculty, University Duesseldorf, Duesseldorf, Germany (K.D.K.)

**Keywords:** endothelial dysfunction, endothelial function, endothelial nitric oxide synthase, microparticles

## Abstract

**Background:**

Microparticles (MPs) are circulating membrane particles of less than a micrometer in diameter shed from endothelial and blood cells. Recent literature suggests that MPs are not just functionally inert cell debris but may possess biological functions and mediate the communication between vascular cells. As a significant proportion of MPs originate from platelets and endothelial cells, we hypothesized that MPs may harbor functional enzymes including an endothelial NO synthase (eNOS).

**Methods and Results:**

Using immunoprecipitation and Western blot analysis, we found that human circulating MPs carry an eNOS. Ca^2+^ and l‐arginine‐dependent NOS activity of crude enzyme extract from MPs was determined by measuring the conversion of [^3^H]‐L‐arginine to [^3^H]‐citrulline and NOS‐dependent nitrite production. NOS‐dependent NO production in intact MPs was assessed by the NO‐specific fluorescent probe MNIP‐Cu. In patients with cardiovascular disease, endothelial dysfunction was associated with an increase in the total number of circulating MPs as well as a significant decrease in the expression and activity of eNOS in MPs. No difference in reactive oxygen species was noted in MPs isolated from either group.

**Conclusions:**

Our data further support the concept that circulating MPs may not only retain phenotypic markers but also preserve the functionality of enzymes of the cells they originate from, including eNOS.

## Introduction

Microparticles (MPs) are shed membrane particles of less than a micrometer in diameter thought to be budded into the circulation from endothelial cells and various blood cells, including platelets, leukocytes, and erythrocytes.^1–3^ Thus, circulating MPs constitute a heterogeneous population of different cellular origins, numbers, size, and antigenic composition. Proposed mechanisms of MP generation include apoptosis and cellular activation by cytokines.^1–3^ MPs circulate in blood from healthy individuals, and their numbers are increased in several cardiovascular diseases and conditions that predispose to cardiovascular disease.^1–3^ MPs have long been considered functionally inert cell debris, and the number of circulating MPs in blood was proposed to be a marker of endothelial damage and platelet activation.^1,3^ More recently, it was appreciated that MPs harbor a number of membrane and cytoplasmatic proteins from the cells they originate from^4–6^ and, therefore, may play a role as a disseminated storage pool of bioactive effectors in intercellular communication mediating effects in cardiovascular physiology and pathophysiology.^1–3,1–8^

The enzyme endothelial nitric oxide synthase (eNOS) and its product nitric oxide (NO) play a central role in the control of vascular homeostasis.^9–10^ A significant proportion of circulating MPs originate from platelets and endothelial cells, which both express eNOS. We hypothesized that MPs may harbor a functional eNOS. Therefore, we analyzed eNOS expression and activity in MPs isolated from healthy individuals, as well as from patients with cardiovascular disease, a condition characterized by impaired eNOS activity and/or expression.

## Methods

### Human Volunteers

Blood was taken from the cubital veins of healthy human volunteers and cardiovascular disease (CVD) patients with documented endothelial dysfunction (Table 1). All subjects gave written informed consent. Procedures were conducted in conformity with the principle embodied in the Declaration of Helsinki and approved by the ethics committee of the Heinrich Heine University. Inclusion was based on a diagnosis of coronary artery disease (CAD), defined as >70% stenosis of ≥1 coronary artery. Exclusion criteria consisted of the presence of heart rhythms other than sinus rhythm, clinical diagnosis of heart failure (NYHA III or IV), recent or current inflammatory condition, renal insufficiency (GFR <60 mL/min), active malignancies within the last year, pre‐ or perimenopausal state, documented noncompliance with medication, and active smoking (>1 cigarette/day).

**Table 1.** Characteristics of Study Subjects

	Healthy Volunteers, Mean±SEM	Patients With CVD, Mean±SEM	*P* Value
Age (y)	27±4	65±5	<0.01
n (Male/female)	12 (8/4)	9 (5/4)	
Body mass index (kg/m^2^)	23.1±0.3	27.3±0.6	<0.01
Protein (g/dL)	7.2±1.5	6.8±1.2	0.651
Glucose (mg/dL)	86±13	144±18	<0.01
Cholesterol (mg/dL)	175±14	211±18	0.0.35
Triglycerides (mg/dL)	108±22	162±24	0.012
High‐density lipoprotein (mg/dL)	62±5	52±8	0.082
Low‐density lipoprotein (mg/dL)	109±19	155±12	0.04
White blood cells (per μL)	6512±882	4264±742	0.052
Polymorph nucleated cells (per μL)	3376±534	3287±502	0.802
Hemoglobin (g/dL)	14±1	12±1	0.079
Lymphocytes (per μL)	1343±134	1593±639	0.532
Monocytes (per μL)	498±54	801±436	0.12
Platelets (×1000/μL)	257±14	302±41	0.310
Mean arterial pressure (mm Hg)	65±5	80±5	0.012
Heart rate (per minute)	62±6	70±8	0.230

CVD indicates cardiovascular disease.

### Materials

Fluoresceine isothiocyanate (FITC)–conjugated monoclonal mouse anti‐human CD235 (glycoforin) antibody was purchased from Miltenyi Biotech (Bergisch Gladbach, Germany), PE‐conjugated mouse anti‐human CD62E came from Beckton Dickinson Pharmingen (Heidelberg, Germany). FITC‐conjugated monoclonal mouse anti‐human CD45 antibody, PC5‐conjugated mouse anti‐human CD41, and PE‐conjugated mouse anti‐human CD144 antibodies and FlowCount fluorospheres were from Beckman Coulter (Krefeld, Germany). Materials for immunoprecipitation, gel electrophoresis, and Western blots were purchased from Invitrogen (Darmstatdt, Germany). Purified monoclonal mouse anti‐human eNOS and purified rabbit anti‐eNOS antiserum were from BD Bioscience (Erembodegem, Belgium). We also used a mouse monoclonal anti‐eNOS (clone NOS‐E1) from Sigma (Deisenhofen, Germany) and phenazine methosulfate (PMS) from Biomol (Enzo Lifescience). l‐N^5^‐(1–iminoethyl)‐ornithine (L‐NIO) and N^G^‐nitro‐l‐arginine‐methyl ester monohydrochloride (L‐NAME) were from Alexis Biochemicals (Loerrach, Germany). Size‐standard microbeads (1 μm) were purchased from Polyscience, Inc. (Eppelheim, Germany). DiD Vybrant Cell, MitoSOX red, and 2′,7′‐dichlorodihydrodiclorofluorescein diacetate (DCF) were purchased from Invitrogen (Karlsruhe, Germany), and chloro[[2,2'‐[1,2‐ethanediylbis[(nitrilo‐κN)methylidyne]]bis[6‐methoxy‐phenolato‐κO]]]‐manganese (EUK‐134) was purchased from Europe BV (Leiden, Netherlands). 4‐Methoxy‐2‐(1H‐naphtho[2,3‐d]imidazol‐2‐yl)phenol (MNIP‐Cu) was synthesized as described.^11^ Unless specified otherwise, chemicals were purchased from Sigma Aldrich (Sigma, Germany).

### Blood Collection and Preparation of Platelet‐Rich Plasma and Platelet‐Free Plasma

Citrated blood (6 mL) was drawn from the cubital vein and processed within 2 hours. Platelet‐rich plasma (PRP) was obtained by centrifugation of whole blood at 300*g* over 15 minutes at room temperature (RT). Platelet‐free plasma (PFP) was obtained by 2 successive centrifugations of PRP at 10 000*g* for 5 minutes at RT. MP pellets and MP‐free plasma samples were obtained by ultracentrifugation of the PFP at 30 000*g* for 90 minutes at 4°C. The protein concentration in the plasma of all blood donors did not differ significantly (Table 1).

### Control of PFP Purification by Laser‐Scanning Microscopy

PRP and PFP were incubated for 30 minutes with DiD, which is a lipophilic carbocyanine dye binding to the phospholipid bilayer of membranes. PFP and PRP were pelleted (30 000*g*, 90 minutes, 4°C), and smears were analyzed 1 to 2 minutes after preparation under a Zeiss LSM 510 confocal laser‐scanning microscope (Carl Zeiss Jena GmbH, Jena, Germany) using a Zeiss Plan Neofluar 63×/1.3 oil DIC objective. DiD was excited with a XeNe laser using a 633‐nm an beam splitter, and fluorescence was recorded with a 670‐nm long‐pass filter. Micrographs were taken at 37°C using an LSM software package (Carl Zeiss Jena GmbH). For each experiment, unstained samples served as the autofluorescence control.

### Characterization of MP Subpopulations by Flow Cytometry

MP subpopulations were discriminated by flow cytometry according to the expression of established surface antigens as described previously.^12^ Briefly, samples were incubated for 30 minutes with fluorochrome‐labeled antibodies or matching isotype controls and analyzed in a Canto II flow cytometer (Beckton Dickinson, Heidelberg, Germany). Microbead standards (1.0 μm) were used to define MPs as <1 μm in diameter. The MP subpopulations were defined as follow: CD41^+^ MPs as platelet‐derived MPs; CD62E^+^, CD144^+^, or CD31^+^/CD41^−^ events as endothelial‐derived MPs; CD235^+^ as erythrocyte‐derived MPs; and CD45^+^ as leukocyte‐derived MPs. The total number of MPs was quantified with flow‐count calibrator beads (20 μL).

### Immunoprecipiatation and Western Blot Analysis

An MP pellet from each individual was lysed by sonication at 4°C and resuspended in 0.2 mL lysis buffer with protease inhibitors (25 mmol/L Tris‐HCl, 150 mmol/L NaCl, 1 mmol/L phenylmethanesulfonyl fluoride, 1 mg/mL aprotinin, 10 mg/mL leupeptin, 1 mmol/L EDTA, 50 mmol/L NaF, 1 mmol/L sodium orthovanadate, 1% Triton‐X [pH 7.6]). Protein concentration was measured using a Biorad DC protein assay kit (Biorad, Munich, Germany). For immunoprecipitation MP lysate (30 μg/μL protein) was incubated for 1 hour at RT with 40 μg of a mouse anti‐human eNOS antibody (BD Bioscience). Immune complexes were isolated by magnetic separation using 50 μL of washed magnetic protein G Dynabeads prepared as recommended by the manufacturer (Invitrogen). Western blot was performed as previously described.^13^ Briefly, 30 μg of MP lysate or the immunocomplexes eluted from the magnetic beads were loaded on 10% NuPAGE Novex Tris/Acetate precast gels (Invitrogen). To control for contamination of plasma proteins in MP preparations, 30 μg of MP‐free plasma (see above) was also loaded into the gels. As reference and normalization of band intensity, we loaded equal amounts (30 μg) of the same human umbilical endothelial cells (HUVECs) lysate in all gels. HUVECs were cultured as described previously.^14^ Proteins were transferred onto PVDF membrane Hybond P (Amersham Biosciences, Munich, Germany). The membrane was stained with a rabbit anti‐eNOS antiserum (1:1000; BD Bioscience) or mouse monoclonal anti‐human eNOS antibody (Sigma Aldrich, Munich, Germany) and with a secondary HRP‐conjugated goat anti‐rabbit or anti‐mouse antibody, respectively (1:5000; Rockland, PA). Equal loading was further controlled by staining duplicate gels by Coomassie Brilliant Blue.

### Conversion of [^3^H]‐L‐Arginine Into [^3^H]‐Citrulline

eNOS activity was determined in 100 μg of MP lysate by measuring the rate of conversion (pmol/min) of [^3^H]‐l‐arginine to [^3^H]‐citrulline as previously described.^15^ The reaction was conducted in a buffer at pH 7.4 containing flavin adenine dinucleotide (2 μmol/L), flavin mononucleotide (2 μmol/L), nicotinamide adenine dinucleotide phosphate (1 mmol/L), tetrahydrobiopterin dihydrochloride (6 μmol/L), Ca^2+^ (75 mmol/L), and calmodulin (0.04 μg/μL), for 2 hours at 37°C and stopped by the addition of cold EDTA (5 mmol/L) in HEPES (50 mmol/L) at pH 5.5. After separation of unreacted [^3^H]‐l‐arginine from [^3^H]‐L‐citrulline with Dowex ion exchange resin, the radioactive signal was detected in a liquid scintillation counter (Wallac 1409, Perkin Elmer, Rodgau, Germany). The amount of sample applied (100 μg MP lysate) was optimized in preliminary experiments by analyzing the rate of [^3^H]‐l‐citrulline formation by a range of 25 to 250 μg of MP lysates. NOS activity was measured in the presence or absence of the specific NOS inhibitor L‐NAME (1 mmol/L) or in the presence of Ca^2+^ chelators EDTA (1 mmol/L) and EGTA (1 mmol/L). The kinetic of the reaction was assessed by measuring [^3^H]‐citrulline equivalents produced at different times (15 to 120 minutes) as indicated compared with the reaction inhibited with L‐NAME. In all experiments, recombinant bovine eNOS was used as a positive control. eNOS activity is reported as picomoles of [^3^H]‐citrulline formed per minute per milligram of total protein.

### NOS‐Derived Nitrite Production

MP lysate was diluted in a 250‐μL reaction buffer containing Tris‐HCL (50 mmol/L), NADPH (1 mmol/L), THB (6 μmol/L), calmodulin (100 nmol/L), and CaCl_2_ (2.5 mmol/L) and incubated for 2 hours at 37°C in the presence or absence of the NOS substrate l‐arginine (3 mmol/L), d‐arginine (3 mmol/L), or the specific NOS inhibitor L‐NIO (3 mmol/L). In all experiments, recombinant bovine eNOS was used as a positive control. The concentration of nitrite as the product of NO oxidation was measured by chemiluminescence.^16^

### Analysis of NO Levels by Laser‐Scanning Microscopy and Flow Cytometry

Intracellular NO levels were detected by staining MPs with MNIP, which was coordinated with Cu(II) to form a stable coordination compound, MNIP‐Cu.^17^ MNIP‐Cu is a cell‐permeable fluorescent probe that reacts rapidly and specifically with NO to generate a blue fluorescence. MNIP was synthesized as previously described.^11^ Stock solutions of MNIP‐Cu were prepared by mixing 1 volume of MNIP (1 mmol/L in dimethyl sulfoxide) and 2 volumes of CuSO_4_ (2.5 mmol/L) and incubated for 30 minutes in the dark.

First, intracellular NO levels were assessed by laser‐scanning microscopy. Platelet‐poor plasma (PPP) was obtained as described for PFP but omitting the second centrifugation step. This led to platelet contamination that we used as a positive control in these experiments. To verify whether NO levels within the circulating MPs contain eNOS activity, PPP was pelleted by centrifugation preincubated with PBS as a control or with the NOS inhibitor L‐NAME for 30 minutes at 37°C and then loaded with MNIP‐Cu (10 μmol/L) for 15 minutes at RT in the dark. PPP smears were analyzed 1 to 2 minutes after preparation under a confocal laser‐scanning microscope. Samples were excited with a UV laser enterprise, and emission was analyzed using a UV/488/543/633‐nm beam splitter and a 350‐nm long‐pass filter. The platelets in the PPP served as a positive control, and MNIP‐Cu‐unloaded samples served as an autofluorescence control.

Second, intracellular NO levels were measured in intact MPs by flow cytometry. PFP was preincubated with PBS as a control or with the NOS inhibitor L‐NAME for 30 minutes at 37°C and then loaded with MNIP‐Cu (10 μmol/L) for 15 minutes at RT in the dark. Samples were analyzed immediately in a Canto II flow cytometer (Beckman Coulter). MNIP‐Cu‐unloaded samples served as an autofluorescence control.

### Detection of Reactive Oxygen Species in MPs by Flow Cytometry

Intraparticular levels of reactive oxygen species (ROS) were measured by the staining of MPs with MitoSox and DCF diacetate. As a control, MPs were treated for 30 minutes at 37°C with 1 mmol/L H_2_O_2_ or with O_2_^−^ generated by mixing 1 mmol/L PMS and 100 μmol/L NADPH. To scavenge ROS, MPs were treated with 80 μmol/L EUK 134, a membrane‐permeable superoxide dismutase/catalase mimetic, where indicated.^18^ MP preparations were loaded for 30 minutes at 37°C with 1 μmol/L DCF or with 1 μmol/L MitoSOX. Probes were then diluted 1:3 in cold PBS and measured in a flow cytometer excited with a 488‐nm argon laser, and fluorescence signals were collected within the PE channel (em 585±42 nm) or the FITC channel (em 530±30 nm), respectively.

### Flow‐Mediated Dilation

Endothelial function was assessed by measuring flow‐mediated dilation of the brachial artery by ultrasound (Sonosite Micromax, Bothell, WA) in combination with an automated analysis system (Brachial Analyzer, Medical Imaging Applications, Iowa City, IO) in a 21°C temperature‐controlled room as described previously.^12^

### Statistical Analyses

Results are expressed as mean±standard error of the mean (SEM). Student *t* tests and repeated‐measures analysis of variance (ANOVA) with the Bonferroni post hoc test were used to evaluate the significance of differences in the mean values between different samples when comparing 2 or >2 samples, respectively. Patient characteristics were analyzed using nonparametric the Mann–Whitney *U* test. *P*<0.05 was considered statistically significant when 2 groups were compared. To avoid inflation of the alpha level, significance levels were adjusted (Bonferroni) when multiple comparisons were computed by dividing the *P* value by the number of post hoc tests performed.

## Results

### Human Circulating MPs Carry an Endothelial Nitric Oxide Synthase

**Figure 1.**
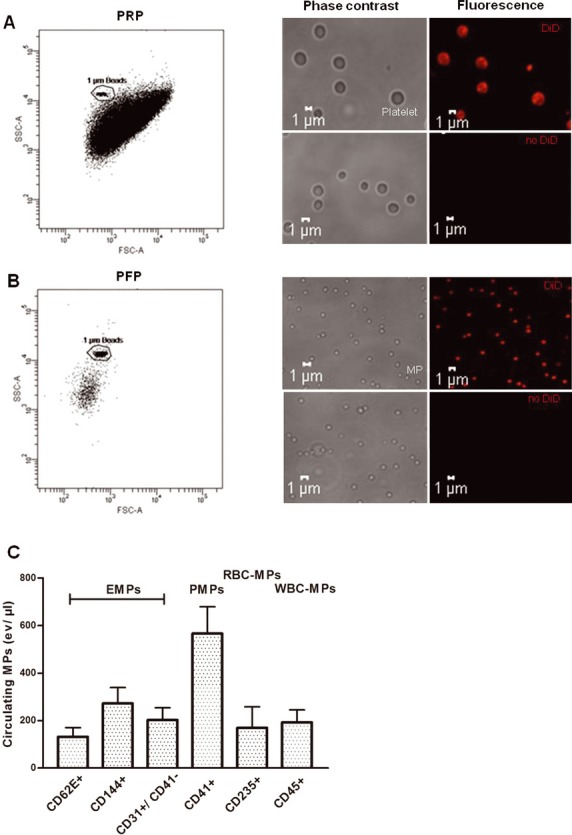
Analysis of composition and morphology of different fractions of human plasma obtained by sequential centrifugation. A, PRP contains a dense population comprising platelets with dimensions >1 μm and MPs with dimensions <1 μm, as shown by flow cytometry (left panel) and phase‐contrast or fluorescence‐based laser‐scanning microscopy (middle and right panels). Membranes were stained with DiD. B, PFP contains MPs only. C, MP subpopulations in PFP were discriminated by flow‐cytometric analysis according to the expression of membrane‐specific antigens: EMPs, PMPs, RBC‐MPs, and WBC‐MPs. Values are mean±SEM. PRP indicates platelet‐rich plasma; PFP, platelet‐free plasma; MPs, microparticles; EMPs, endothelial‐derived microparticles; PMPs, platelet‐derived microparticles; RBC‐MPs, erythrocyte‐derived micoparticles; WBC‐MPs, leukocyte‐derived microparticles.

Removal of platelet contamination is crucial for proteomic analysis of circulating MPs in plasma. We purified MPs by sequential centrifugation of human plasma. Analysis of the different fractions by flow cytometry and laser‐scanning microscopy revealed that PRP contained both platelets with a size >1 μm, ranging from 1.5 to 3 μm in diameter, and MPs with a size <1 μm (Figure 1A). PFP did not contain any platelets (Figure 1B). The major subpopulations of MPs (Figure 1C) were identified to be of platelet origin (CD41^+^, 40%), closely followed by endothelial origin (CD41^−^/CD31^+^, 25%; CD144^+^, 28%; CD62e^+^, 5%). Other subpopulations were erythrocyte‐derived (CD235^+^, 10%) and leukocyte‐derived (CD45^+^, 8%) MPs.

**Figure 2.**
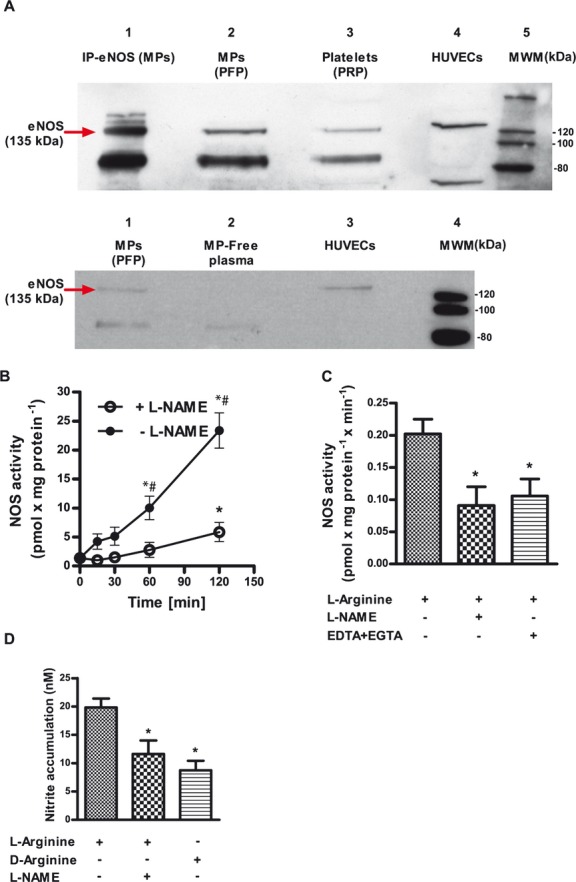
MPs express a functional eNOS. A, Western blot analysis of eNOS obtained by immunoprecipitation (IP) from human MPs, crude extracts of circulating MPs, and platelets, as well as HUVECs as a control (top) and MPs, MP‐free plasma, and HUVECs (bottom). B, Crude MP lysate converted [^3^H]‐l‐arginine to [^3^H]‐l‐citrulline in a time‐dependent fashion. *P<0.0125 compared with baseline, #*P*<0.0125 compared with L‐NAME (2B). C, NOS activity is inhibited by the NOS inhibitor L‐NAME and by the Ca^2+^ chelators EDTA and EGTA. D, NOS‐dependent nitrite accumulation was substrate dependent (n=6). *P<0.025 compared with l‐arginine only. *P* values represent Bonferroni‐corrected significance levels. MPs indicates microparticles; eNOS, endothelial nitric oxide synthase; PRP, platelet‐rich plasma; PFP, platelet‐free plasma; HUVEC, human umbilical vein endothelial cell; MWM, molecular weight marker; L‐NAME, N^G^‐nitro‐l‐arginine‐methyl ester monohydrochloride.

We found that MPs express an eNOS (Figure 2A), as demonstrated by both immunoprecipitation of eNOS with a mouse monoclonal anti‐eNOS antibody from PFP (Figure 2A, lane 1) or Western blot analysis of a crude MP lysate (Figure 2A, lane 2). The same band at an approximate molecular weight of 135 kDa was also present in lysate from platelets and from human endothelial cells (Figure 2A, lanes 3+4). The specificity of the band was further confirmed by staining with a rabbit anti‐eNOS antibody or a mouse monoclonal anti‐eNOS antibody directed against different epitopes. No eNOS was detected in MP‐free plasma (Figure 2A, bottom gel).

### eNOS Protein in Circulating MPs Is Active and Produces NO

Enzymatic activity of eNOS was determined in MP lysate by analyzing the conversion of [^3^H]‐l‐arginine to [^3^H]‐l‐citrulline in the presence of NADPH, FAD, FMN, Ca^2+^, and calmodulin. We measured a significant increase in [^3^H]‐citrulline production over time, which was inhibited by the addition of the specific NOS inhibitor L‐NAME (Figure 2B). Ca^2+^‐chelation by EDTA and EGTA also strongly impaired [^3^H]‐l‐citrulline production (Figure 2C).

NOS activity was also determined by measuring NOS‐dependent nitrite accumulation in the presence of l‐arginine and enzymatic cofactors. In the presence of l‐arginine and Ca^2+^/calmodulin, the measured nitrite accumulation was 19.8±1.8 nmol/L within 120 minutes of incubation. If the inactive substrate d‐arginine was added instead of l‐arginine or after the addition of the NOS inhibitor L‐NAME, nitrite accumulation was significantly impaired (8.8±1.6 and 11.6±2.4 nmol/L, respectively; Figure 2D).

**Figure 3.**
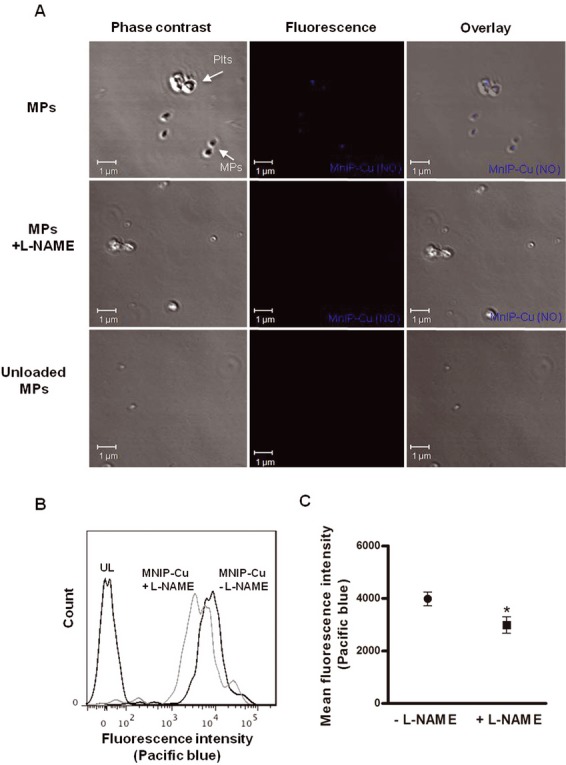
Direct detection of NO in human circulating MPs. MPs loaded with the NO fluorescent probe MNIP‐Cu were highly fluorescent (CTRL), as measured by laser‐scanning microscopy (A) and flow cytometry (n=10; B+C). The addition of the specific NOS inhibitor L‐NAME decreased fluorescence. Unloaded MPs (UL) were used as a control for autofluorescence. *P<0.05 compared with samples without L‐NAME. NO indicates nitric oxide; MPs, microparticles; NOS, nitric oxide synthase; L‐NAME, N^G^‐nitro‐l‐arginine‐methyl ester monohydrochloride; MNIP‐Cu, 4‐methoxy‐2‐(1H‐naphtho[2,3‐d]imidazol‐2‐yl)phenol.

We also found that intact MPs produced NOS‐derived NO. A significant increase in intraparticular fluorescence was achieved using a NO‐specific membrane‐permeable fluorescent probe (MNIP‐Cu). The platelet contamination within PPP was used as a positive control in these experiments, as platelets are known to express an active eNOS producing NO. Laser‐scanning microscopy revealed a blue fluorescence signal in MPs and platelets loaded with the NO probe MNIP‐Cu (Figure 3A, upper panels). Pretreatment with the NOS inhibitor L‐NAME significantly blunted fluorescent activity, comparable to the unloaded control MPs (Figure 3A, middle panels). The same observations were confirmed by flow cytometry (Figure 3B and 3C). Unloaded samples were analyzed to control for autofluorescence (Figure 3A, bottom panel, and Figure 3B, first peak).

### Levels of MP eNOS Protein and Activity in Patients With Endothelial Dysfunction

**Figure 4.**
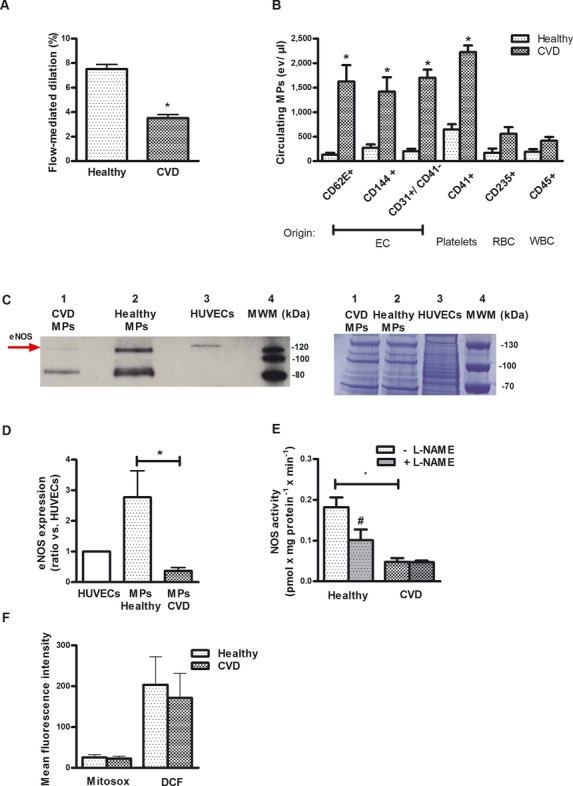
The MPs' eNOS activity is decreased in patients with endothelial dysfunction. Patients with cardiovascular disease (CVD) exhibited decreased endothelial function as assessed by flow‐mediated vasodilation (A) and increased MPs levels (especially endothelial cell– and platelet‐derived MPs) in plasma (B) compared with healthy subjects. C+D, MPs from these patients carry less eNOS protein compared with healthy controls. C, Western blot analysis, representative of n=5 independent gels (left); protein bands stained with colloidal Coomassie Brilliant Blue, confirming equal gel loading between samples (right). D, Densitometry band intensity was normalized vs a standard HUVEC lysate and was quantified using Image J software^©^. *P<0.017 compared with healthy subjects. E, NOS activity in MPs from CVD (l‐citrulline synthesis) was decreased compared with healthy controls. F, Mean fluorescence intensity of MPs labeled with MitoSox, a probe for O_2_^−^, or DCF, a probe for ROS, did not significantly differ between groups (measured by flow cytometry). *P<0.05 compared with healthy subjects. Values are mean±SEM. MPs indicates microparticles; eNOS, endothelial nitric oxide synthase; EC, endothelial cell; RBC, red blood cell; WBC, white blood cell; HUVEC, human umbilical vein endothelial cell; L‐NAME, N^G^‐nitro‐l‐arginine‐methyl ester monohydrochloride; DCF, 2′,7′‐dichlorodihydrodiclorofluorescein diacetate; ROS, reactive oxygen species.

Defective endothelial and platelet NO synthesis represents a major feature of endothelial dysfunction in cardiovascular disease (CVD). To verify whether their circulating “offspring” also show similar features, we investigated eNOS activity and NOS expression in MPs of patients with CVD and documented endothelial dysfunction as compared with healthy volunteers (see Table 1 for characteristics). Indicative of endothelial dysfunction, patients exhibited impaired flow‐mediated vasodilation (7.5±0.4% versus 3.5±0.3%, *P*=0.001; Figure 4A). The total number of circulating MPs was increased in the plasma of CVD patients compared with healthy controls (Figure 4B). This was mainly driven by an increase in endothelial cell– and platelet‐derived MPs. In a subgroup of the study population, we analyzed eNOS expression and activity (n=11, healthy; n=7, CAD). In MPs circulating in the plasma of CVD patients, we detected significantly reduced levels of eNOS protein (Figure 4C) compared with in healthy volunteers. Figure 4D shows the densitometric analysis. Band intensity was normalized for the intensity of the eNOS band in HUVECs. Equal loading was confirmed by staining a duplicate gel with Coomassie Brilliant Blue.

Of interest, the NOS‐dependent conversion of [^3^H]‐l‐arginine to [^3^H]‐l‐citrulline was largely abolished (Figure 4E), as also demonstrated by the lack of a measurable effect of the NOS inhibitor L‐NAME.

**Figure 5.**
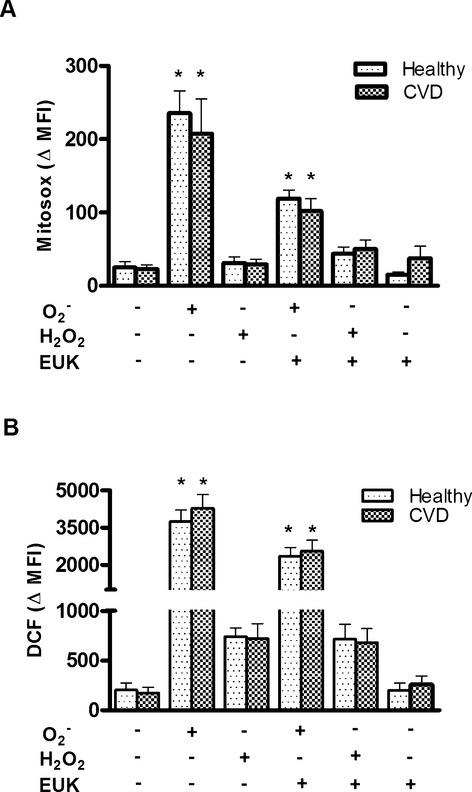
ROS levels in MPs from healthy subjects and patients with CVD. Mean fluorescence intensity of (A) MitoSox, a probe for O_2_^−^, and (B) DCF, a probe for ROS, did not significantly differ between groups. MPs were treated with PBS as a control, with an O_2_^−^‐generating system, with H_2_O_2_ or with the superoxide dismutase mimic EUK. MPs were stained with MitoSox (n=5) or with DCF diacetate (n=5) or were left unlabeled as an autofluorescence control. Probes were analyzed in a flow cytometer. *P<0.05 compared with the control. Values are mean±SEM. MPs indicates microparticles; ROS, reactive oxygen species; DCF, 2′,7′‐dichlorodihydrodiclorofluorescein diacetate; CVD, cardiovascular disease. MFI, mean fluorescence intensity; EUK‐134, chloro[[2,2'‐[1,2‐ethanediylbis[(nitrilo‐κN)methylidyne]]bis[6‐methoxy‐phenolato‐κO]]]‐manganese.

We also compared superoxide generation in MPs from the patients with cardiovascular diseases and from healthy volunteers. Our data showed that the mean fluorescence intensity of MitoSox, a probe for O_2_^−^, as well as of DCF, a probe for ROS, measured by flow cytometry, did not significantly differ between the groups (Figure 4F). As a positive control, we treated MPs with O_2_^−^ or with H_2_O_2_. This strongly increased intracellular fluorescence activity of MitoSox as well as of DCF. The addition of EUK 134, a cell membrane–permeable ROS scavenger, decreased the intracellular fluorescence activity of Mitosox and DCF (Figure 5). In summary, our data suggest that the decrease in NOS activity was mainly a result of decreased protein levels in MPs from CVD patients compared with healthy individuals, whereas ROS levels were unchanged.

## Discussion

We have demonstrated that circulating MPs express an active eNOS and produce NO. In patients with cardiovascular diseases, impaired endothelium‐dependent vasodilation was associated with decreased eNOS expression and activity in circulating MPs.

To the best of our knowledge, we have shown for the first time that human circulating MPs express a functionally active eNOS protein. Previously, Leroyer et al^19^ identified an eNOS protein in MPs derived from cultured mouse endothelial cells. In addition, our data have shown that eNOS in circulating MPs is not just a nonfunctional residual protein of the original cell, but rather an enzyme retaining its catalytic function. Thus, we have shown that NOS is capable of converting l‐arginine to l‐citrulline and of producing NO in a substrate‐ and Ca^2+^‐dependent fashion. In intact MPs, we also demonstrated NOS‐dependent NO production by detecting MNIP‐Cu‐derived fluorescence activity, which was inhibited by preincubating with a NOS inhibitor. Taken together, we have shown that MPs express an active eNOS that produces NO within the MPs.

We were not able to distinguish between the subpopulations of MPs by means of their contribution to overall eNOS. As shown in our present and in previous studies,^2^ in healthy individuals, the major subpopulation of circulating MPs is derived from platelets and endothelial cells, both of which have eNOS localized to the membrane.^20–26^ Nevertheless, leukocytes and erythrocytes also express an eNOS^27–30^ and may also release MPs into circulation.

Here, we have shown for the first time that MPs isolated from patients with cardiovascular disease and endothelial dysfunction show less eNOS expression and activity than healthy volunteers' MPs. Moreover, in these patients an increase in the total amount of circulating MPs was measured, confirming previous research.^1–3^

Defective endothelial NO synthesis represents a major feature of endothelial dysfunction and vascular disease.^31–32^ It was previously demonstrated in cardiovascular disease that endothelial cells^31,33^ and platelets^34^ exhibit decreased eNOS protein expression and NO release. Thus, our results show that under these pathological conditions, not only the parent cells of the predominant MP subpopulations are dysfunctional regarding eNOS expression and eNOS activity, but also their circulating “offspring.” Therefore, eNOS activity of MPs may be a useful available readout of endothelial NOS activity in vivo. A global decrease in eNOS activity in endothelium and platelets as well as in the MPs originating from them may indicate global eNOS dysfunction in cardiovascular disease. Future studies in larger patient populations might address the questions of how eNOS expression is distributed among the MP subpopulations and whether MPs are able to transfer their “enzymatic legacy” to other cells in health and disease.

The consequences of decreased eNOS protein expression and NO release are not limited to the vessel wall,^31,33^ but may also involve blood cells known to express this enzyme, including platelets,^34^ endothelial progenitor cells,^14^ or even circulating MPs, as shown in the present study. Considering the central role played by endothelial and blood cell eNOS in vascular homeostasis, it is also tempting to speculate that the eNOS activity of MPs may directly participate in this equilibrium, providing a further circulating source of NO. Future studies will aid further understanding of the (patho)physiological role of circulating eNOS in MPs.

Taken together, our data support the concept that MPs may not only retain phenotypic markers but also preserve the expression and functionality of enzymes of the cells they originate from, including eNOS, and may mediate and transport their physiological functions. In cardiovascular disease, an increase in endothelium‐derived MP subpopulations accompanied by a decrease in MPs' eNOS activity may not merely be an index of a dysfunctional endothelium, but may also contribute to disturbed NO bioavailability and hence play a role in the pathophysiology of cardiovascular disease.
